# Practical strategies for reducing suicide risk among depressed adults

**DOI:** 10.1192/j.eurpsy.2021.1329

**Published:** 2021-08-13

**Authors:** J. Overholser, S. Hernandez

**Affiliations:** Psychology & Psychiatry, Case Western Reserve University, Cleveland, United States of America

## Abstract

**Introduction:**

Suicide remains a major problem throughout society. Unfortunately, recommendations for the treatment of suicidal clients are often presented at a general level, without providing adequate detail that could guide the practicing clinician.

**Objectives:**

The review explains three main strategies that can be used to helpr educe the risk of suicide among depressed adults.

**Methods:**

Method: The review identified three themes derives from an integration of 30+ years of clinical experience working with depressed outpatients combined with a comprehensive review of recent journal articles on depression and suicide.

**Results:**

First, clients may become suicidal when they focus on unfortunate events from their recent or distant past, resulting in tendencies for rumination and guilt. Therapy can help clients cultivate an attitude of contentment, promoting self-forgiveness and a sense of accomplishment. Second, suicidal clients often focus on their current struggles, frequently involving financial problems, interpersonal conflict, and social isolation. Therapy can help clients to embrace life through planned activities, reconnecting with loved ones, and repairing damaged relationships. Third, clients may struggle because of hopeless views of their future, feeling trapped in a desperate situation with no possible solution. Therapy can help clients look to the future with a more optimist attitude and a sense of control.

**Conclusions:**

Clients can learn to search for realistic solutions to their problems, developing a renewed sense of optimism and empowerment. The risk of suicide can be reduced when therapy helps clients reduce guilt and worthlessness, increase meaningful social bonds, and instill realistic hope for the future.

**Conflict of interest:**

No significant relationships.

